# Comparison of CT and CMR for detection and quantification of carotid artery calcification: the Rotterdam Study

**DOI:** 10.1186/s12968-017-0340-z

**Published:** 2017-03-06

**Authors:** Blerim Mujaj, Andrés M. Arias Lorza, Arna van Engelen, Marleen de Bruijne, Oscar H. Franco, Aad van der Lugt, Meike W. Vernooij, Daniel Bos

**Affiliations:** 1 Department of Epidemiology, Erasmus MC, University Medical Center, Office Na 2824k, PO Box 2040, 3000 CA Rotterdam, The Netherlands; 2 Biomedical Imaging Group Rotterdam, Departments of Medical Informatics, Radiology, and Nuclear Medicine, Erasmus MC University Medical Center, Rotterdam, The Netherlands; 3 Department of Radiology and Nuclear Medicine, Erasmus MC University Medical Center, Rotterdam, The Netherlands; 4 Department of Computer Science, University of Copenhagen, Copenhagen, Denmark; 5 Department of Clinical Epidemiology, Harvard TH Chan School of Public Health, Boston, USA

**Keywords:** CT, CMR, Carotid artery, Atherosclerosis, Calcification, Stroke

## Abstract

**Background:**

Carotid artery atherosclerosis is an important risk factor for stroke. As such, quantitative imaging of carotid artery calcification, as a proxy of atherosclerosis, has become a cornerstone of current stroke research. Yet, population-based data comparing the computed tomography (CT) and cardiovascular magnetic resonance (CMR) for the detection and quantification of calcification remain scarce.

**Methods:**

A total of 684 participants from the population-based Rotterdam Study underwent both a CT and CMR of the carotid artery bifurcation to quantify the amount of carotid artery calcification (mean interscan interval: 4.9 ± 1.2 years). We investigated the correlation between the amount of calcification measured on CT and CMR using Spearman’s correlation coefficient, Bland-Altman plots, and linear regression. In addition, using logistic regression modeling, we assessed the association of CT and CMR based calcification volumes with a history of stroke.

**Results:**

We found a strong correlation between CT and CMR based calcification volumes (Spearman’s correlation coefficient:0.86, p-value ≤0.01). Bland-Altman analyses showed a good agreement, though CT based calcification volumes were systematically larger. Finally, calcification volume assessed with either imaging modality was associated with a history of stroke with similar effect estimates (odds ratio (OR) per 1-SD increase in calcification volume: 1.52 (95% CI:1.00;2.30) for CT, and 1.47 (95% CI:1.01;2.14) for CMR.

**Conclusion:**

CT based and CMR based volumes of carotid artery calcification are highly correlated, but CMR based calcification is systematically smaller than those obtained with CT. Despite this difference, both provide comparable information with regard to a history of stroke.

**Electronic supplementary material:**

The online version of this article (doi:10.1186/s12968-017-0340-z) contains supplementary material, which is available to authorized users.

## Background

Atherosclerosis located at the bifurcation of the carotid artery is an important risk factor for stroke [1–5]. As such, quantification of the severity of carotid atherosclerosis has become an increasingly important topic in stroke research. Multiple non-invasive imaging techniques, including ultrasound, computed tomography (CT), and cardiovascular magnetic resonance (CMR), are currently available to obtain measures of the extent of atherosclerosis [6]. An important advantage of CT and CMR is that both modalities offer possibilities for detailed characterization and quantification of the atherosclerotic plaque [7]. The mostly studied characteristic of the atherosclerotic plaque is calcification, given that it is one of the most prominent plaque characteristics and represents a reliable marker of the underlying plaque burden [8]. For the visualization of calcification, non-contrast CT is acknowledged to be superior to any other imaging modality [9]. Yet, thanks to rapid technological advances, non-contrast CMR now also allows for the detection and quantification of calcification in the atherosclerotic plaque [10] and has the major advantage over CT that it does not involve radiation exposure. Moreover, with CMR it is possible to visualize additional plaque characteristics such as intraplaque hemorrhage or lipid-rich necrotic core which provide unique additional information on the disease. Despite these potential advantages of CMR, it remains unclear whether calcification volumes obtained with CMR are comparable to those measured with CT. Against this background, we set out to quantify and compare CT-based and CMR-based carotid artery calcification in terms of absolute volumes and with respect to the history of stroke as a relevant clinical outcome, in participants from the population-based Rotterdam Study.

## Methods

### Setting

This study was carried out within the framework of the Rotterdam Study, a prospective population-based study among middle-aged and elderly persons [11]. Between 2003 and 2006, all participants that visited the research center were invited to undergo multi-detector computed tomography (MDCT) to quantify vascular calcification in multiple vessels, including the carotid artery bifurcation [12]. In total 2,524 participants were scanned.

From October 2007 onwards, carotid CMR was incorporated in the Rotterdam Study. Between 2007 and 2012, we invited 2,666 participants to undergo CMR of the carotid arteries to study atherosclerotic disease. These participants were selected on the basis of the presence of atherosclerosis in at least one carotid artery on ultrasound examination (defined as intima-media thickness >2.0 mm in one or both carotid arteries), which is regularly performed in all Rotterdam Study participants. In total 1,982 participants underwent carotid CMR. From these 1,982, 808 participants had also undergone a CT-examination. Due to image artifacts or low image quality (*n* = 31, or errors in the CMR registration process needed for analysis (*n* = 93) 124 participants were excluded, leaving 684 participants with usable CT and CMR data for the current study. The mean time interval between CT scan and CMR scan was 4.9 years (standard deviation 1.2 years).

### Assessment of CT-based calcification

**Fig. 1:**
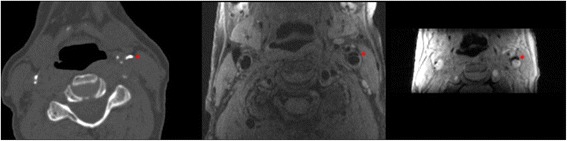
Example of calcification in the left carotid artery bifurcation (indicated by the *red star*) on CT (*left* image) and on CMR (*middle* image; PDw-FSE-BB sequence, and *right* image; magnitude image of the 3D-phase contrast sequence)

We performed a non-enhanced CT-examination (16-or 64-slice MDCT Somatom Sensation, Siemens, Forchheim, Germany) that reached from the aortic arch to the intracranial vasculature, to visualize calcification in the extracranial carotid arteries. The detailed information regarding the scan protocol is described elsewhere [12]. In short, the following scan parameters were used: 16 x 0.75 mm collimation, 120 kVp, 100 effective mAs, and 0.5 s rotation time, with a normalized pitch of 1. Images were reconstructed with an effective slice width of 1 mm, a reconstruction interval of 0.5 mm, and a medium sharp convolution kernel [12]. Calcification in the extra-cranial carotid artery was measured bilaterally within three centimeters proximal and distal of the bifurcation and was automatically quantified with dedicated commercially available software (syngo calcium scoring, Siemens, Germany) [12]. Calcification volumes in both carotid arteries were expressed in cubic millimeters (mm^3^) [13] (Fig. 1).

### Assessment of CMR-based calcification

CMR of the carotid arteries was performed on a single 1.5-T scanner (GE Healthcare, Milwaukee, WI, USA) with a dedicated bilateral phased-array surface coil (Machnet, Eelde, The Netherlands). The high-resolution images were obtained using a standardized protocol [14]. First, both carotids were identified by means of two-dimensional (2D) time-of-flight MR angiography. Second, high-resolution CMR sequences were planned to image the carotid bifurcations on both sides. These sequences consisted of four 2D sequences in the axial plane, namely a proton density weighted (PDw)-fast spin echo (FSE)-black blood (BB) sequence (in-plane resolution 130/160 x 130/128 = 0.8 x 1 cm); a PDw-FSE-BB with an increased in-plane resolution (in-plane resolution 130/224 x 130/160 = 0.5 x 0.8 cm); a PDw-echo planar image (EPI) sequence (in-plane resolution 130/160 x 70/160 = 0.8 x 0.4 cm); and a T2w-EPI sequence (in-plane resolution 130/160 x 70/160 = 0.8 x 0.4 cm). Additionally, we performed two 3D sequences, namely a 3D-T1w-gradient echo (GRE) sequence (in-plane resolution 180/192 x 180/180 = 0.9 x 1 cm), and a 3D phased-contrast MR angiography (in-plane resolution 180/256 x 180/128 = 0.7 x 1.4 cm) (Additional file 1: Table S3). The total scanning time was approximately 30 min [14]. Calcification was evaluated bilaterally within three centimeters proximal and distal of the bifurcation [12]. All calcification measurements on CMR were performed by one trained physician under the supervision of an experienced neuroradiologist. We performed an intra- and inter-observer reproducibility analysis on a random set of 30 CMR examinations. The intra- and inter-agreement was very good [Cohens’ Kappa : 0.91 (95% CI 0.82–0.99) and 0.94 (95% CI 0.86–0.99)], respectively. We defined calcification as a hypointense region in the plaque on all sequences. We manually annotated and segmented calcification in all plaques using a standardized approach. First, we pre-processed all images using a method that has been described extensively before [15]. This starts with a bias correction to reduce the intensity inhomogeneity characteristic in CMR [15]. Subsequently, the carotid artery in all images was rigidly registered to the black-blood image space using the Elastix tool [15]. For the registration of the sequences, a Region Of Interest (ROI) around the artery in black-blood was used. This ROI was obtained semi-automatically by uniformly growing an extracted carotid artery centerline, which requires three marked seed points at the common, internal and external parts of the artery [15]. Then calcification was manually delineated in every consecutive slice using an annotation tool developed in Mevislab (MeVisLab, MeVis Medical Solutions AG). Fourth, the total volume of calcification was calculated by counting the number of voxels within the annotated areas and multiplying this by the voxel volume (Fig. 1). This provided volumes of calcification in cubic millimeters.

### Assessment of history of stroke

At study entry, all participants were interviewed and a history of stroke was assessed. Moreover, after enrollment, all participants are continuously followed for the occurrence of stroke [16]. All potential stroke events were reviewed by research physicians and verified by an experienced stroke neurologist [17]. At the time of CT scan, 38 participants had suffered a prior stroke [16].

### Statistical analysis

Due to skewed distributions of the calcification data, we used natural log (Ln) transformed values after we added 1.0 mm^3^ to the non-transformed data in order to deal with calcification scores of zero (Ln (calcification volume +1.0 mm^3^)) [16]. Our analysis strategy consisted of four steps. First, we investigated the correlation of CT-based calcification volumes with CMR-based calcification volumes using Spearman’s correlation coefficient. Second, we used linear regression to assess the relation between CT-based and CMR-based calcification volumes while adjusting for the time interval between the scans. Given the substantial time interval between the CT and CMR examinations, we furthermore performed a sensitivity analysis in which we analyzed the correlation between CT-based and CMR-based calcification volumes only for those persons with an interval equal or less than 3 years (*n* = 128). We performed post-hoc sensitivity analysis while adjusting for CT-scanner type also.

Third, we assessed the agreement between CT-based and CMR-based calcification volumes using a Bland-Altman analysis. Fourth, as a proof-of-principle, we investigated the association of CT-based and CMR-based calcification volumes (per 1-SD increase) related with a history of stroke using logistic regression while adjusting for age, sex and the time interval between CT and CMR, and studied whether the results were comparable for both modalities All analyses were carried out using IBM SPSS Statistics version 21 (International Business Machines Corporation, Armonk, New York).

## Results

Table 1 shows the baseline characteristics of the study population. The mean age of participants at the time of CT examination was 68.1 years (SD: 6.1 years). There were 41.5% female participants. We found no calcification in 60 participants (8.8%). There were no instances in which calcification was found on either CT or CMR and not on the other modality. The mean Ln-transformed calcification volume for CT was 3.98 mm^3^ (SD: 1.86 mm^3^), and 2.70 mm^3^ (SD: 1.36 mm^3^) for CMR.

**Table 1:** Baseline characteristics of study participants

Sample size	684
Woman	41.5%
Age, years at CT scan	68.8 ± 6.1
Age, years at CMR scan	74.2 ± 6.1
CT calcification volumes, mm^3a^	3.98 ± 1.87^a^
CMR calcification volumes, mm^3a^	2.70 ± 1.37^a^
Smoking (current)	40.2%
Systolic blood pressure (mm/Hg)	146.81 ± 19.46
Diastolic blood pressure (mm/Hg)	79.84 ± 10.85
Diabetes Mellitus	13.3%
Serum total cholesterol (mmol/L)	5.6 ± 0.9
HDL cholesterol (mmol/L)	1.4 ± 0.3
Antihypertensive medication use	37.7%
Statin medication use	31.1%
Stroke events	5.6%

Values are means with standard deviations for continuous variables and percentages for dichotomous or categorical variables

*Abbreviation*: *CT* computed tomography, *HDL* high-density lipoprotein, *CMR* cardiovascular magnetic resonance

^a^Ln-transformed volumes (Ln(calcification volume + 1 mm^3^))

**Fig. 2:**
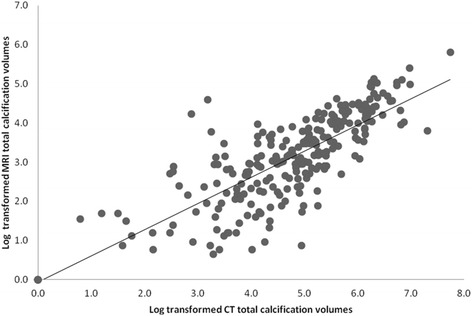
Scatter plot of Ln-transformed CT-based and CMR-based calcification volumes, indicating a positive correlation between both detected and quantified calcification volumes

We found a strong correlation between CT and CMR calcification volumes (Spearman’s correlation coefficient:0.86) (Fig. 2, Additional file 1: Table S1, and Additional file 1: Table S2). This correlation was similar when we investigated the left and right side separately (Additional file 1: Table S1). After performing linear regression with adjustment for the time interval between the CT and CMR scan, the prominent relation between CT-based and CMR-based calcification volumes remained present [beta per 1-SD increase in CT-based calcification volume: 0.65 (95% confidence interval (CI): 0.63;0.68)]. After performing the analyses in those persons with a time interval between the scans of less or equal to 3 years, the association between CT-based and CMR-based calcification volumes was similar [beta per 1-SD increase in CT-based calcification volume: 0.65 (95% CI: 0.58;0.72)]. Adjustment for CT-scanner type did not influence the results (data not shown).

**Fig. 3:**
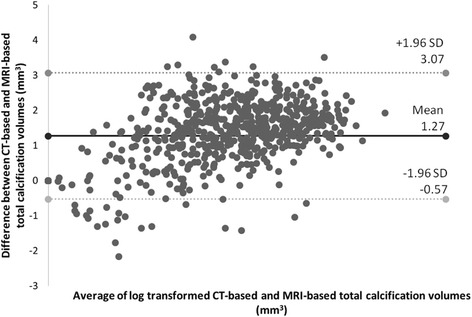
Bland-Altman plot of the difference of CT-based and CMR-based Ln-transformed total calcification volumes, with a mean absolute difference (*bold continues line*) and 95% confidence interval of mean differences (*dashed lines*)

Figure 3 shows the Bland-Altman plot for the relation between the absolute differences in Ln-transformed calcification volumes and the mean of the two measurements of 1.27 mm^3^ (standard deviation: 0.92). We found that the CT-based calcification volumes were consistently larger than those obtained from CMR.

When investigating the relationship between calcification and a history of stroke, we found that both CT-based and CMR-based calcification volumes were associated with a history of stroke [CT - odds ratio per 1-SD increase: 1.52 (95% CI: 1.00; 2.30), CMR – odds ratio per 1-SD increase: 1.47 (95% CI: 1.01; 2.14)] (Table 2).

**Table 2:** Association of calcification volumes with stroke

	Odds ratio (95% CI)	*p*-value
Model 1		
CT calcification volumes	1.63 (1.09–2.46)	0.01
CMR calcification volumes	1.55 (1.07–2.24)	0.01
Model 2		
CT calcification volumes	1.52 (1.00–2.30)	0.04
CMR calcification volumes	1.47 (1.01–2.14)	0.04

Model 1 - scan time difference

Model 2 –adjusted for age, sex and scan time difference. Values represent odd ratios with 95% CI per 1 standard deviation increase in calcification volumes

*Abbreviation*: *CT* computed tomography, *CMR* cardiovascular magnetic resonance

## Discussion

In this large population-based sample of persons with subclinical atherosclerosis, we found that CT-based and CMR-based volumes of carotid artery calcification are highly correlated, but CMR-based calcification is systematically smaller than those obtained with CT. Despite this difference, both provide comparable information with regard to a history of stroke.

We found that CT-based and CMR-based calcification volumes were highly correlated. Yet, we also found that the volumes measured with CMR were systematically smaller than those measured on CT. This was especially interesting in light of the fact that the CMR was performed on average 4 years later than the CT. Given that our scanning protocol on CT was specifically designed for the visualization of vascular calcification combined with that CT is currently the gold standard for the assessment calcification, it is likely that with CMR the amount of calcification is systematically underestimated [6]. The reason for this could the differences between CT-based and CMR-based calcification volume may be explained by differences in image analysis to a certain extent. Additionally, differences in spatial resolution between CT and CMR might be a potential explanation for this difference. In this light, it is important to note that CT images were analyzed automatically using dedicated commercially available software, whilst CMR images were analyzed manually for the presence and amount of calcification. To our knowledge, there are no studies that have compared CT and CMR on the detection and quantification of carotid artery using a non-invasive population-based approach. Previous research performed on the comparison between CT and CMR in 50 patients with recent TIA or minor stroke, demonstrated a correlation between CT-based and CMR-based calcification volumes of the only *p*: 0.55 [18]. We demonstrate that with the use of dedicated CMR-multi-sequences for the detection of calcification the correlation between CT-based and CMR-based calcification volume is substantially improved. Finally, another important topic to consider with regard to the difference between CT and CMR is the blooming effect of calcifications which is known to occur on CT [19]. Especially for calcifications with very high Hounsfield units, a gradient over multiple adjacent pixels is necessary to reach a low Hounsfield unit. This effect may lead to slight overestimation of the calcification area. On the other hand, CMR is known to underestimate the amount of calcification, because a certain amount of calcification is required before the MR-signal disappears. In this context, it is important to acknowledge that possible micro-calcifications in the atherosclerotic plaque may be missed [20].

As a proof of principle, we investigated the association of CT-based and CMR-based calcification with a history of stroke and found that both related to this outcome with comparable effect estimates. We chose history of stroke because the relationship between carotid artery calcification and stroke has been well-established [16, 21, 22]. Importantly, despite the fact that CMR systematically underestimates the amount of calcification compared to CT, we found comparable risk estimates for CT-based and CMR-based calcification volumes with respect to a history of stroke. This suggests that when assessing clinical outcomes, the value of CMR-based calcification is similar to that of CT.

Our findings have implications that should be considered in the choice for CMR or CT for the assessment of vascular calcification. First, while assessing atherosclerosis with CMR it is directly possible to visualize other plaque characteristics in addition to calcification, including intra-plaque hemorrhage and lipid-rich necrotic core which provide unique additional information on the disease. Second, CMR has the major advantage over CT that it does not involve harmful radiation exposure. Third, the systematic underestimation of calcification on CMR may pose a problem, specifically in situations where one is particularly interested in the exact amount of calcification. Fourth, drawbacks of CMR, in general, are its absolute contraindications (i.e. metal objects in the body), and the fact that CMR is more time-consuming, more expensive and less widely available than CT. Taken together, the pros and cons of both imaging modalities should be carefully considered for all research and clinical applications involving the assessment of vascular calcification.

The strengths of our study include the relatively large sample size of community-dwelling individuals, all with varying degrees of carotid atherosclerosis, and the standardized assessment of calcification volumes on both modalities. Yet, some limitations should also be taken into account of which the first is the time interval between the CT scan and the CMR scan, with a mean interval of 4.9 years. We acknowledge that the interscan interval represents a potential limitation of the current study and that during this interval there may have been slight changes in plaque composition. Yet, we would like to emphasize that in all instances the CT-scan was made before the CMR-scan and that calcification is a plaque component that generally remains present and shows only very slow progression over time [23, 24]. Therefore, it seems unlikely that the amount of calcification at the time of CMR would differ substantially from that at the time of the CT. This is further supported by the fact that adjustment for the time interval did not change the results; and secondly by our finding that CMR volumes were consistently estimated somewhat smaller than CT volumes, whereas a large influence of the time interval would induce an opposite difference. Another potential limitation is that we used two types of MDCT scanners (16-slice and 64-slice) to assess calcification. Yet, adjustment for scanner-type did not change the association.

## Conclusion

In summary, CT-based and CMR-based volumes of carotid artery calcification are highly correlated, but CMR-based calcification is systematically smaller than those obtained with CT. Despite this difference, both provide comparable information with regard to a history of stroke.

## Additional file

Additional file 1: Table S1–S3. (Relation between calcification volume on CT and CMR), (Relation between calcification volume on CT and CMR, between the subjects with <3 years and >3 years difference on CT and CMR scans). (Parameters of the CMR Protocol). (DOCX 18 kb)

## Abbreviations

CI: Confidence interval
CMR: Cardiovascular magnetic resonance
CT: Computed tomography
ERGO: Erasmus Rotterdam Gezondheid Onderzoek
MDCT: Multi detector computed tomography
OR: Odds ratio
SD: Standard deviation
TIA: Transient ischemic attack

## References

[1] Mozaffarian D, Benjamin EJ, Go AS, Arnett DK, Blaha MJ, Cushman M, Das SR, de Ferranti S, Despres JP, Fullerton HJ (2016). Executive Summary: Heart Disease and Stroke Statistics-2016 Update: A Report From the American Heart Association. Circulation.

[2] Kwee RM (2010). Systematic review on the association between calcification in carotid plaques and clinical ischemic symptoms. J Vasc Surg.

[3] Donnan GA, Fisher M, Macleod M, Davis SM (2008). Stroke. Lancet.

[4] Lusis AJ (2000). Atherosclerosis. Nature.

[5] Libby P, Ridker PM, Hansson GK (2011). Progress and challenges in translating the biology of atherosclerosis. Nature.

[6] Owen DR, Lindsay AC, Choudhury RP, Fayad ZA (2011). Imaging of atherosclerosis. Annu Rev Med.

[7] Golledge J, Siew DA (2008). Identifying the carotid 'high risk' plaque: is it still a riddle wrapped up in an enigma?. Eur J Vasc Endovasc Surg.

[8] Nandalur KR, Baskurt E, Hagspiel KD, Finch M, Phillips CD, Bollampally SR, Kramer CM (2006). Carotid artery calcification on CT may independently predict stroke risk. AJR Am J Roentgenol.

[9] Chalela JA (2009). Evaluating the carotid plaque: going beyond stenosis. Cerebrovasc Dis.

[10] Truijman MT, Kooi ME, van Dijk AC, de Rotte AA, van der Kolk AG, Liem MI, Schreuder FH, Boersma E, Mess WH, van Oostenbrugge RJ (2014). Plaque At RISK (PARISK): prospective multicenter study to improve diagnosis of high-risk carotid plaques. Int J Stroke.

[11] Hofman A, Brusselle GG, Darwish Murad S, van Duijn CM, Franco OH, Goedegebure A, Ikram MA, Klaver CC, Nijsten TE, Peeters RP (2015). The Rotterdam Study: 2016 objectives and design update. Eur J Epidemiol.

[12] Odink AE, van der Lugt A, Hofman A, Hunink MG, Breteler MM, Krestin GP, Witteman JC (2007). Association between calcification in the coronary arteries, aortic arch and carotid arteries: the Rotterdam study. Atherosclerosis.

[13] van den Bouwhuijsen QJ, Bos D, Ikram MA, Hofman A, Krestin GP, Franco OH, van der Lugt A, Vernooij MW (2015). Coexistence of Calcification, Intraplaque Hemorrhage and Lipid Core within the Asymptomatic Atherosclerotic Carotid Plaque: The Rotterdam Study. Cerebrovasc Dis.

[14] van den Bouwhuijsen QJ, Vernooij MW, Hofman A, Krestin GP, van der Lugt A, Witteman JC (2012). Determinants of magnetic resonance imaging detected carotid plaque components: the Rotterdam Study. Eur Heart J.

[15] Arias-Lorza AM, Petersen J, van Engelen A, Selwaness M, van der Lugt A, Niessen WJ, de Bruijne M (2016). Carotid Artery Wall Segmentation in Multispectral MRI by Coupled Optimal Surface Graph Cuts. IEEE Trans Med Imaging.

[16] Bos D, Portegies ML, van der Lugt A, Bos MJ, Koudstaal PJ, Hofman A, Krestin GP, Franco OH, Vernooij MW, Ikram MA (2014). Intracranial carotid artery atherosclerosis and the risk of stroke in whites: the Rotterdam Study. JAMA Neurol.

[17] Wieberdink RG, Poels MM, Vernooij MW, Koudstaal PJ, Hofman A, van der Lugt A, Breteler MM, Ikram MA (2011). Serum lipid levels and the risk of intracerebral hemorrhage: the Rotterdam Study. Arterioscler Thromb Vasc Biol.

[18] Kwee RM, Teule GJ, van Oostenbrugge RJ, Mess WH, Prins MH, van der Geest RJ, Ter Berg JW, Franke CL, Korten AG, Meems BJ (2009). Multimodality imaging of carotid artery plaques: 18 F-fluoro-2-deoxyglucose positron emission tomography, computed tomography, and magnetic resonance imaging. Stroke.

[19] de Weert TT, Ouhlous M, Meijering E, Zondervan PE, Hendriks JM, van Sambeek MR, Dippel DW, van der Lugt A (2006). In vivo characterization and quantification of atherosclerotic carotid plaque components with multidetector computed tomography and histopathological correlation. Arterioscler Thromb Vasc Biol.

[20] Baheza RA, Welch EB, Gochberg DF, Sanders M, Harvey S, Gore JC, Yankeelov TE (2015). Detection of microcalcifications by characteristic magnetic susceptibility effects using MR phase image cross-correlation analysis. Med Phys.

[21] Bos D, Ikram MA, Elias-Smale SE, Krestin GP, Hofman A, Witteman JC, van der Lugt A, Vernooij MW (2011). Calcification in major vessel beds relates to vascular brain disease. Arterioscler Thromb Vasc Biol.

[22] Rennenberg RJ, Kessels AG, Schurgers LJ, van Engelshoven JM, de Leeuw PW, Kroon AA (2009). Vascular calcifications as a marker of increased cardiovascular risk: a meta-analysis. Vasc Health Risk Manag.

[23] van Gils MJ, Bodde MC, Cremers LG, Dippel DW, van der Lugt A (2013). Determinants of calcification growth in atherosclerotic carotid arteries; a serial multi-detector CT angiography study. Atherosclerosis.

[24] van Gils MJ, Vukadinovic D, van Dijk AC, Dippel DW, Niessen WJ, van der Lugt A (2012). Carotid atherosclerotic plaque progression and change in plaque composition over time: a 5-year follow-up study using serial CT angiography. AJNR Am J Neuroradiol.

