# Supplementary material for: Comparison of CT and CMR for detection and quantification of carotid artery calcification: the Rotterdam Study
Source: J Cardiovasc Magn Reson. 2017 Mar 6;19:28. doi: 10.1186/s12968-017-0340-z (PMC5338077; doi:10.1186/s12968-017-0340-z)
Supplement: Additional file 1: Table S1–S3. — (Relation between calcification volume on CT and CMR), (Relation between calcification volume on CT and CMR, between the subjects with <3 years and >3 years difference on CT and CMR scans). (Parameters of the CMR Protocol). (DOCX 18 kb) [file 12968_2017_340_MOESM1_ESM.docx]

| **Additional file 1: Table S1** Relation between calcification volume on CT and MRI | | | |
| --- | --- | --- | --- |
|  | MRI left carotid | MRI right carotid | MRI total volumes |
| CT left carotid | 0.77 |  |  |
| CT right carotid |  | 0.78 |  |
| CT total volumes |  |  | 0.86 |
| Correlation is significant at the 0.01 level (2-tailed).  Abbreviation: CT = computed tomography, MRI = magnetic resonance imaging. | | | |

| **Additional file 1: Table S2** Relation between calcification volume on CT and MRI, between the subjects with <3 years and >3 years difference on CT and MRI scans. | | | |
| --- | --- | --- | --- |
|  | MRI <3 years  n=128 | MRI >3  n=556 | MRI total volumes  n=684 |
| CT <3 years | 0.79 |  |  |
| CT >3 years |  | 0.87 |  |
| CT total volumes |  |  | 0.86 |
| Correlation is significant at the 0.01 level (2-tailed).  Abbreviation: CT = computed tomography, MRI = magnetic resonance imaging. | | | |

| **Additional file 1: Table S3** Parameters of the MRI Protocol | | | | | | |
| --- | --- | --- | --- | --- | --- | --- |
|  | 2-D | | | | 3-D^*^ | |
|  | FSE-BB | | EPI | | PC-MRA | GRE |
|  | PDw | | PDw | T2w |  | T1w |
|  | Thin slice | High Resolution |  |  |  |  |
| TE, (ms) | 9.8 | 12.7 | 24.3 | 60 | 4.3 | 1.8 |
| TR, (ms) | 4800 | 2000 | 12000 | 12000 | 13 | 15.7 |
| ETL | 6 | 4 | - | - | - | - |
| Field of View, (cm) | 13x13 | 13x13 | 13x7 | 13x7 | 18x18 | 18x18 |
| Matrix | 160x128 | 224x160 | 160x160 | 160x160 | 256x128 | 192x180 |
| Slice thickness, (mm) | 0·9 | 1·2 | 1·2 | 1·2 | 1·0/0·5^†^ | 1·0/0·5^†^ |
| No. of slices | 51 | 19 | 41 | 41 | 26/52 | 124/248 |
| NEX | 2 | 3 | 20 | 25 | 1 | 1 |
| Scan time, (min.sec) | 3·36 | 4·04 | 4·00 | 5·00 | 6·13 | 6·02 |

FSE-BB indicates Fast Spin Echo Black Blood; EPI, Echo Planar Imaging; PC-MRA, Phased-Contrast Magnetic Resonance Angiography; GRE, Gradient Recalled Echo; PD, proton density; TR, repetition time; TE, echo time; ETL, echo train length; NEX, No. of excitations; 2-D, two-dimensional; 3-D, three-dimensional.

* = Axial images are reconstructed from the 3-D volume

† = Images are interpolated from 1·0 mm to 0·5 mm
